# NAPG mutation in family members with hereditary hemorrhagic telangiectasia in China

**DOI:** 10.1186/s12890-021-01524-4

**Published:** 2021-06-10

**Authors:** Yu Xu, Yong-Biao Zhang, Li-Jun Liang, Jia-Li Tian, Jin-Ming Lin, Pan-Pan Wang, Rong-Hui Li, Ming-Liang Gu, Zhan-Cheng Gao

**Affiliations:** 1 Department of Pulmonary and Critical Care Medicine, Peking University People’s Hospital, Beijing, 100044 People’s Republic of China; 2 Interdisciplinary Innovation Institute of Medicine and Engineering, Beijing Advanced Innovation Center for Big Data-Based Precision Medicine, School of Biological Science and Medical Engineering, Beihang University, Beijing, 100109 People’s Republic of China; 3 CAS Key Laboratory of Genome Sciences and Information, Beijing Institute of Genomics, Chinese Academy of Sciences, Beijing, 100101 People’s Republic of China; 4 Joint Laboratory for Translational Medicine Research, Beijing Institute of Genomics, Chinese Academy of Sciences and Liaocheng People’s Hospital, Liaocheng City, 252000 Shandong Province People’s Republic of China

**Keywords:** Hereditary hemorrhagic telangiectasia, Whole-exome sequencing, NAPG

## Abstract

**Background:**

Hereditary hemorrhagic telangiectasia (HHT) is a disease characterized by arteriovenous malformations in the skin and mucous membranes. We enrolled a large pedigree comprising 32 living members, and screened for mutations responsible for HHT.

**Methods:**

We performed whole-exome sequencing to identify novel mutations in the pedigree after excluding three previously reported HHT-related genes using Sanger sequencing. We then performed in silico functional analysis of candidate mutations that were obtained using a variant filtering strategy to identify mutations responsible for HHT.

**Results:**

After screening the HHT-related genes, activin A receptor-like type 1 (*ACVRL1*), endoglin (*ENG*), and SMAD family member 4 (*SMAD4*), we did not detect any co-segregated mutations in this pedigree. Whole-exome sequencing analysis of 7 members and Sanger sequencing analysis of 16 additional members identified a mutation (c.784A > G) in the NSF attachment protein gamma (*NAPG*) gene that co-segregated with the disease. Functional prediction showed that the mutation was deleterious and might change the conformational stability of the NAPG protein.

**Conclusions:**

*NAPG* c.784A > G may potentially lead to HHT. These results expand the current understanding of the genetic contributions to HHT pathogenesis.

**Supplementary Information:**

The online version contains supplementary material available at 10.1186/s12890-021-01524-4.

## Background

Hereditary hemorrhagic telangiectasia (HHT) is a group of related disorders inherited in an autosomal dominant fashion, characterized by the development of arteriovenous malformations (AVMs) in the skin and mucous membranes. AVMs may occur anywhere in the body but are most apparent on the lips, tongue, face, fingertips, and the nasal, buccal, and gastrointestinal mucosa [1]. AVMs may also be found in internal organs such as the brain, lung, and liver. Due to the thin walls and proximity to the skin or mucosal surfaces, vessels are prone to rupture and bleeding with minimal injury. Epistaxis and mucocutaneous telangiectasias are the most common clinical manifestations [2, 3].

HHT is thought to affect people regardless of ethnicity and has been reported in many nations [2, 4, 5], with an estimated prevalence ranging from 1 in 5000 to 8000 [4]. Three genes, namely activin A receptor-like type 1 (*ACVRL1*), endoglin (*ENG*), and SMAD family member 4 (*SMAD4*), have been reported as causative for HHT and were confirmed in Chinese cohorts. Using genetic screening, Jia et al. confirmed the presence of a mutation in *ACVRL1* that led to a change in the protein structure [6]. Two other studies found that mutations in *ACVRL1* and *ENG* were predisposing genetic factors in Chinese Han patients [7, 8]. In this study, we analyzed an HHT pedigree and performed genetic screening, finding no mutations in the three known HHT-related genes. This suggested that other gene mutations may also be involved in HHT. Therefore, we performed whole-exome sequencing (WES) analysis of selected members from the pedigree to identify new candidate gene mutations associated with HHT.

## Methods

## Ethics statement

Informed consent was obtained from patients or their guardians. The study was approved by the Ethics Committee of the Peking University People’s Hospital, and complied with the requirements of the Declaration of Helsinki and ICH-GCP.

### Study subjects and laboratory analysis

The proband was admitted for hemoptysis, and his bronchoscopy results revealed hemorrhagic telangiectasia in the tracheal and bronchial mucosa. The patient disclosed that he had epilepsy and was prone to epistaxis when he was young. The occurrence of epistaxis and epilepsy was also observed in other family members. We collected blood samples from 23 family members and determined the platelet count (UnicelDxH 800, Coulter Cellular Analysis System, FL, USA) and platelet activity (Chrono-Log, Corporation, MODEL 700, PA, USA). We also collected the medical histories of these family members to identify the HHT clinical subtype.

### Screening previously reported HHT-related genes

We designed primers for all exons of *ACVRL1*, *ENG*, and *SMAD4* and sequenced the DNA of the proband and his family members to detect mutations in these genes. In brief, primer sets for PCR and sequencing were designed using Primer-Premier 5.0 (PREMIER Biosoft International, Palo Alto, CA, USA) (Additional file 1: Table 1). All PCR was performed using the touchdown method, and PCR products were purified using an AcroPrep 384-well Filter Plate 30 K (Pall, Port Washington, NY, USA). Sequencing reactions were conducted using Applied Biosystems BigDye Terminator chemistry, and the products were resolved on an ABI Prism 3730xl DNA Analyzer (Applied Biosystems). Sequence trace files were analyzed using Phred/Phrap/Polyphred/Consed software (University of Washington, Seattle, WA, USA). The base-quality value threshold was set to 20 in Phred (i.e., a 99% probability that the base is accurate).

### WES and variant calling

Within the analyzed HHT pedigree, four affected individuals, one obligate carrier, and two normal individuals (one of them the spouse of a family member) were selected for WES. The Sure Select Human All Exon 70 Mb kit (Agilent Technologies, Santa Clara, CA, USA) was used to capture the whole exome. Paired-end sequencing with a 150-bp read length was conducted for each sample on a HiSeq X Ten sequencer (Illumina, San Diego, CA, USA). All reads were mapped to the human reference genome (hg19) using BWA (version 0.7.5a-r405). PCR duplication was removed using Picard (version 1.92). GATK (version 3.7) was used to call the variants. We used Pindel and cn.MOPS software to detect structural variants and copy number variations, respectively [9, 10]. Variants were then annotated using Seattle Seq Annotation 138, sequentially filtered, and assessed according to the following methodology described by Sun et al. [11]: (1) removal of ariants with a global minor allele frequency (MAF) greater than 0.01 in the dbSNP 138 or 1000 Genomes Project databases; (2) retention of variants consistent with a model of dominant disease transmission; (3) removal of variants with MAF greater than 0.01 in the National Heart, Lung, and Blood Institute (NHLBI) Exome Sequencing Project (ESP), Exome Aggregation Consortium (ExAC), and International Haplotype Map (HapMap) databases; (4) retention of only possible loss-of-function variants, including missense, frame-shift, near splice-site, and stop-gain/stop-loss variants; (5) retention of the variants that passed manual confirmation using the IGV package; and (6) retention of variants that are predicted as deleterious (PolyPhen score > 0.6) or near splice-site variants.

### Genotyping of all collected sample using Sanger sequencing

We designed primers for candidate genes obtained from the abovementioned process (Additional file 1: Table 2) and sequenced DNA collected from 16 other pedigree members. The processes of primer design, PCR amplification, DNA sequencing, and variant calling are the same as those described in the subsection “Screening previously reported HHT-related genes”.

### Structure prediction of NSF attachment protein gamma (NAPG) protein

To evaluate the structure of NAPG and the effect of the *NAPG* p.M262V mutation on protein conformation, we used the following software packages: PSIPRED for secondary structure prediction, Swiss-model for tertiary structure prediction, and Swiss-Pdb Viewer 4.1 for tertiary structure display and manipulation. In Swiss-Pdb Viewer, the following parameters were used: minimum energy, residues within 6 Å to p.M262V, secondary structure as ribbon format, colorful secondary structure by types, and computing H-bonds and van der Waals forces.

## Results

### Clinical characteristics

**Fig. 1:**
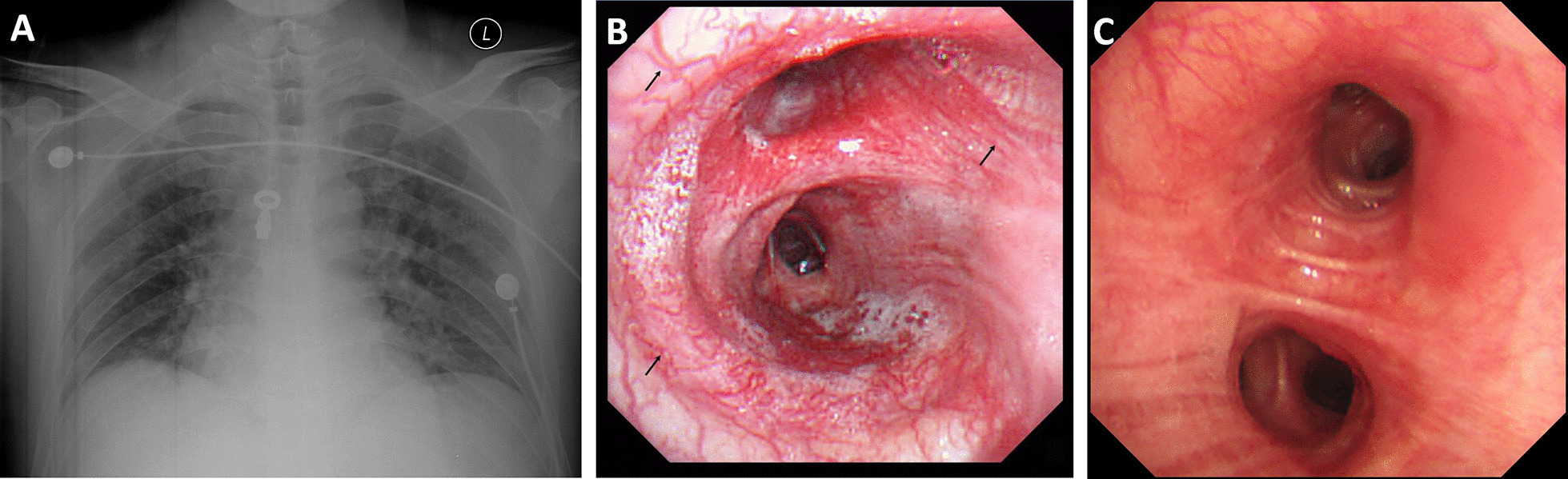
Imaging and bronchoscope results of the proband (III-11). **a** Normal the proband’s chest X-ray. **b** The left main bronchus, with telangiectasia evident under the bronchial mucosa (arrow). **c** Another patient’s bronchoscopy, showing a normal capillary vessel under the bronchial mucosa

**Fig. 2:**
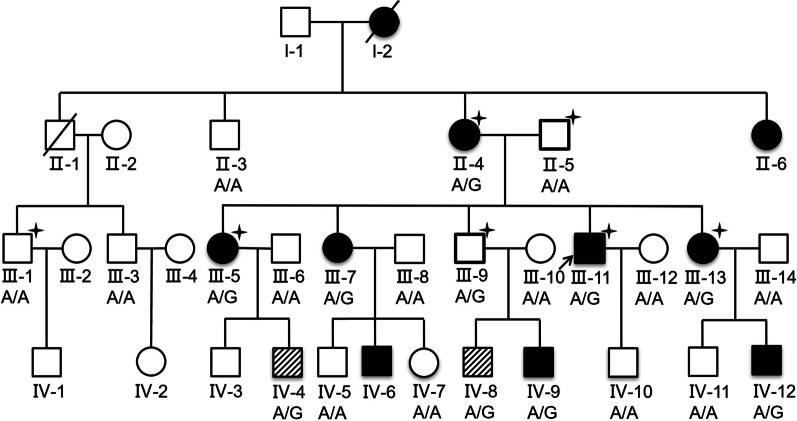
Phenotypes and *NAPG* genotypes of the HHT pedigree. Squares are used for males, circles for females, and slashes for the deceased. III-11 is the proband (arrow). Black symbols show members with both epistaxis and epilepsy. Shadow symbols show members with epistaxis only. Whole-exome sequencing was conducted on individuals labeled with a star (upper right star). A/A or A/G indicates the genotype of *NAPG* c.784A > G

The proband of the HHT family was born in 1967 and is from Shanxi province of China. The patient was admitted for hemoptysis, and a chest radiograph did not find any abnormality in his lungs. His laboratory tests, such as platelet counts and coagulation function, were normal. To determine the bleeding site causing hemoptysis, we performed bronchoscopy on this patient. Results showed hemorrhagic telangiectasia in the tracheal and bronchial mucosa (Fig. 1). The patient told us that he had seizures, and there was a higher incidence rate of epistaxis and seizure in his family members than in other residents, especially when the family members were infants or teenagers. Some family members were admitted to the hospital for seizure and were diagnosed with epilepsy. Therefore, we obtained clinical data from the whole family (32 living members), and the pedigree is shown in Fig. 2 (the proband is III-11). Blood samples from 23 family members were collected, 9 of which exhibited epistaxis and 7 were diagnosed with epilepsy when they were infants. Their platelet-related laboratory tests of platelets were normal, with mean platelet counts of 266.30 × 10^9^ ± 65.57 × 10^9^/L (normal range: 150–350 × 10^9^/L) and platelet activity of 71.80 ± 11.85% (normal range: 50–150%).

### WES and HHT candidate gene determination

**Fig. 3:**
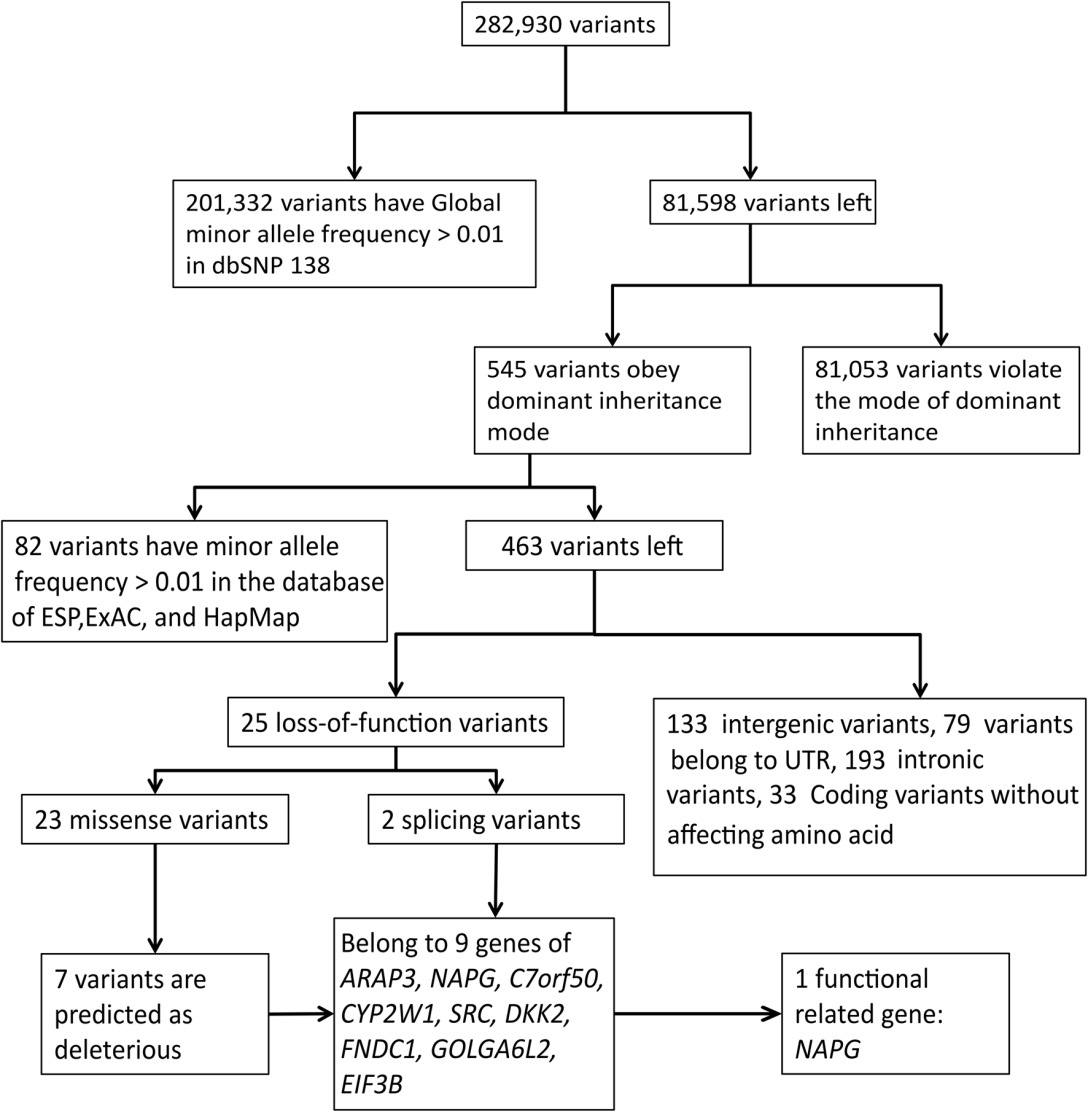
Workflow for variant prioritization

To explore the genetic mechanism underlying this HHT pedigree, we performed WES analysis of four diagnosed patients (II-4, III-5, III-11, and III-13; Fig. 2), one obligate carrier (III-9; Fig. 2), and two unaffected individuals (II-5, and III-1; Fig. 2). The exome sequencing showed a mean coverage of 43- to 109-fold (Additional file 1: Table 3). In total, 282,930 variants, including 282,447 single nucleotide variants and small indels, 25 structural variants, and 458 copy number variants were identified. As 201,332 of these variants had a global MAF larger than 0.01 in the dbSNP138 database, those variants were removed from the variants library. Of the remaining 81,598 variants, 545 were retained for adhering to the autosomal dominant inheritance mode suggested by the pedigree, i.e., mutations carried by the four diagnosed patients and one obligate carrier but not by the two unaffected individuals were retained. By further cross-checking with the NHLBI ESP, ExAC, and HapMap databases, we eliminated 82 variants that had MAFs larger than 0.01. Subsequently, we focused on variants that were missense, frame-shift, splicing, or stop-gain/stop-loss variants and obtained 25 possible loss-of-function variants. After predicting the harmful effects of these 25 variants, we selected 7 deleterious variants and 2 splicing variants as HHT candidates (Fig. 3). These nine variants were located in *ARAP3*, *NAPG*, *C7orf50*, *CYP2W1*, *SRC*, *DKK2*, *FNDC1*, *GOLGA6L2*, and *EIF3B* (Table 1).

**Table 1:** The 9 candidates for HHT after variants filtering

CHR	Position^a^	Ref/Alt	SNP	GenBank transcript ID	Function	Amino Acid changes	cDNA changes	PolyPhen	CADD Score	Gene	MAF in GnomAD
5	141,036,099	C/T	rs75953884	NM_022481	missense	ARG,HIS	c.3761G > A	0.632	16.68	*ARAP3*	0.000733
18	10,549,082	A/G	novel	NM_003826	missense	MET,VAL	c.784A > G	0.957	5.54	*NAPG*	NA
7	1,049,698	T/C	rs568083670	NM_001134395	missense	LYS,GLU	c.211A > G	0.996	15.11	*C7orf50*	0.000201
7	1,026,318	C/T	rs369538884	NM_017781	missense	ARG,HIS	c.704G > A	0.997	12.47	*CYP2W1*	0.000008
20	36,022,675	C/T	rs146865960	NM_005417	missense	THR,MET	c.548C > T	0.997	13.53	*SRC*	0.000008
4	107,845,819	G/A	rs146683175	NM_014421	missense	HIS,TYR	c.412C > T	0.999	28.70	*DKK2*	0.001529
6	159,618,528	T/C	rs374967242	NM_032532	missense	TRP,ARG	c.175 T > C	1	16.42	*FNDC1*	0.000325
15	23,685,007	TCTCCTCCTGCCCC/T	rs139919701	NM_001304388.2	frameshift-near-splice	none	c.2602_2614delGGGGCAGGAGGAGA	-	-	*GOLGA6L2*	NA
7	2,405,952	G/A	rs147656962	NM_001037283	synonymous-near-splice	ARG, ARG	c.1158G > A	-	0.95	*EIF3B*	0.002907

^a^Physical position according human reference genome hg19

Ref/Alt: Reference allele/Alternative allele, CHR:chromosome, SNP: single nucleotide polymorphism, MAF: minor allele frequency

We firstly performed Sanger sequencing of DNA of an additional eight members of the pedigree (II-3, III-3, III-6, III-7, III-8, III-10, III-12, and III-14; Fig. 2) at the SNP sites of the nine candidate genes, and found that only *NAPG* c.784A > G co-segregate with the disease (Fig. 2). To further verify this result, the DNA of eight fourth-generation family members was subjected to Sanger sequencing (IV-4, IV-5, IV-7, IV-8, IV-9, IV-10, IV-11, and IV-12; Fig. 2). We found that *NAPG* c.784A > G was well segregated within this generation (present in all four patients), suggesting its strong correlation with the disease. In addition, after searching the GeneMatcher database [12], we found that *NAPG* has not been previously reported.

### In silico modeling of the *NAPG*c.784A > G mutation

**Fig. 4:**
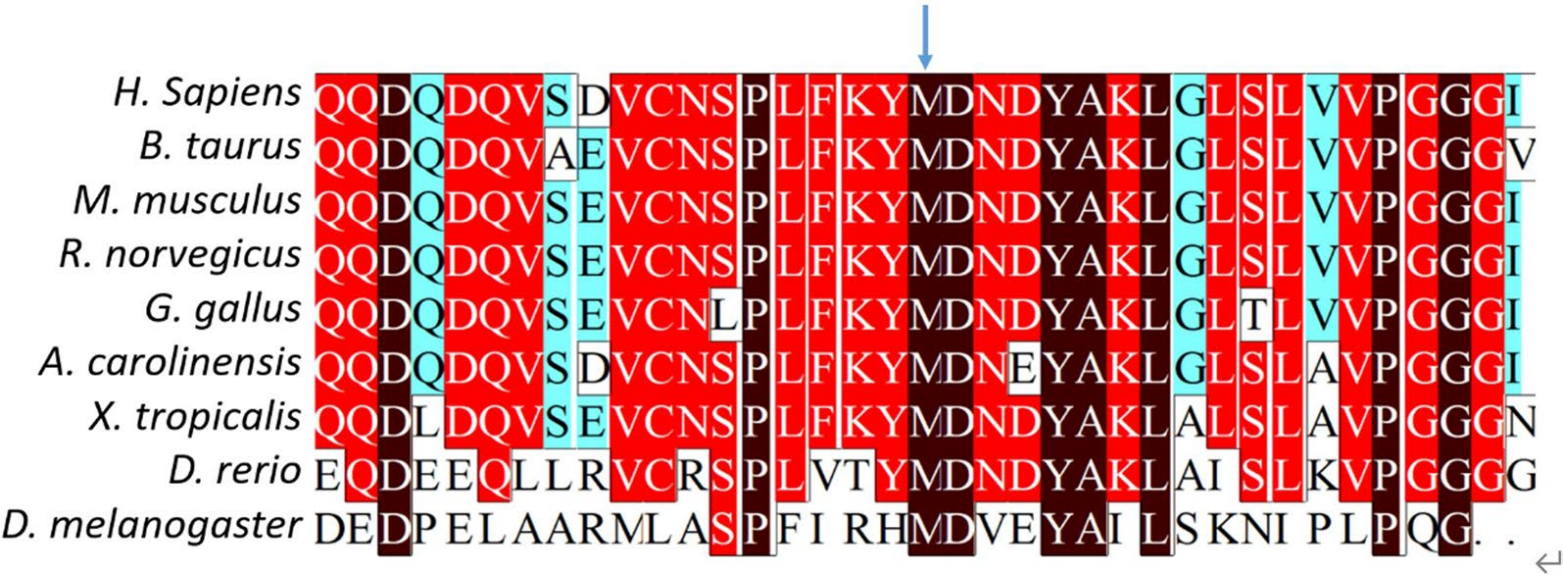
Alignment and conservation of residues among NAPG orthologs. Black highlighting indicates 100% amino acid homology across species; red and cyan highlighting indicates 75% and 50% homology, respectively. The arrow indicates the highly conserved MET262

**Fig. 5:**
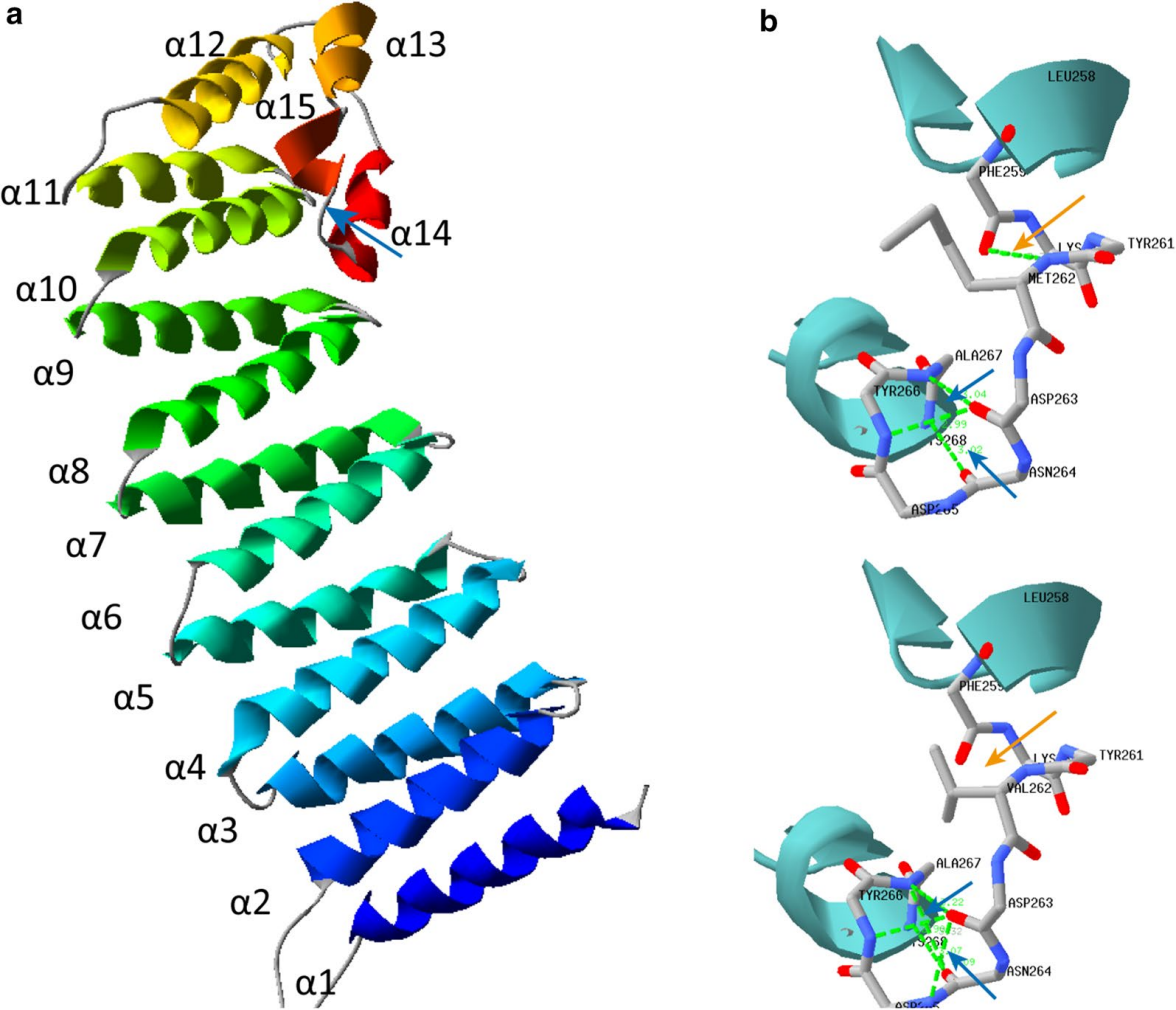
Tertiary structure prediction of *NAPG* and hydrogen bond changes after MET262VAL mutation. A, A cartoon representation of the human NAPG structure in the rainbow coloring scheme from the N-terminal (blue) to the Carboxyl-terminal (red); the blue arrow indicates MET262VAL. B, Hydrogen bonds in wild-type NAPG. C, Hydrogen bonds in NAPG p.262 M > V. Green dotted lines indicate hydrogen bonds. Numbers indicate hydrogen bond distances. The orange arrow indicates a hydrogen bond loss between PHE259 and VAL262. The blue arrows indicate new hydrogen bond formations between ASP263 and ASP265, and between ASN264 and ALA267

*NAPG* c.784A > G is located on chromosome 18, resulting in an amino acid change from methionine to valine. MET262 is highly conserved in NAPG across species, even between humans and fruit flies (Fig. 4). Although there is no reported structure for the human NAPG protein, the zebrafish NAPG (75% homologous to the human protein) has been revealed as an elongated all-helix protein composed of 15 α-helices, with four helix hair-pins on the N-terminal and one helix bundle on the Carboxyl-terminal [13]. Based on the homology, the protein structure prediction software produced a similar all-helix protein structure for human NAPG. MET262 resided immediately before α15 close to the C-terminal, which happens to be a ligand (sulfate ion) pocket, as indicated in the zebrafish NAPG structure (Fig. 5a). Given the potential of the MET262VAL mutation to alter the interaction between protein and ligand, we performed in silico modeling of this mutation. MET262 forms a hydrogen bond with PHE259, which is lost after the mutation (Fig. 5b, c), and this could explain why the VAL residue was predicted to have a higher energy than the MET one in the protein (Additional file 1: Table 4). Moreover, the MET262VAL variant changed the spatial conformation of the proximate ASP263, resulting in the formation of two new hydrogen bonds between ASP263 and ASP265 as well as between ASN264 and ALA267 (Fig. 5c). These changes could sabotage the active site of the protein. Therefore, MET262VAL might have changed the conformation and stability of the C-terminal, which is responsible for N-ethylmaleimide-sensitive fusion protein binding [14], leading to NAPG dysfunction.

## Discussion

HHT is a rare disease known as an autosomal-dominantly inherited vascular malformation syndrome, characterized by telangiectasias and AVMs. Two genes in the transforming growth factor-beta (TGF-β) signaling pathway, *ENG* and *ACVRL1*, were discovered almost two decades ago, and mutations in these genes have been reported to cause up to 85% of HHT cases. Besides, 2% of patients with HHT carry *SMAD4* mutations [2].

So far, there are no data on the epidemiology of HHT in China. In our study, while genetic screening of the family revealed no mutations in the *ENG*, *ACVRL1*, or *SMAD4* genes, we identified an A/G mutation in *NAPG*. *NAPG* encodes a gamma-soluble NSF attachment protein that mediates platelet exocytosis and controls membrane fusion events [15]. The mutation identified in *NAPG* caused an amino acid change from methionine to valine, which is predicted to change the stability of the C-terminal of the protein. As Koseoglu et al. showed that granule exocytosis is required for platelet spreading [16]. We speculate that changes in stability of NAPG may cause a deficiency in platelet spreading.

We assessed platelet counts and activity in the family members that carried *NAPG* mutation, and observed that only one individual (IV-12, an 8-year-old boy) showed an increased platelet number. All tested family members presented normal platelet activity. These results indicate that *NAPG* mutation did not influence platelet function in vitro. It is well known that the early formation of blood cell plugs that seal a disrupted endothelial barrier is a repair mechanism that, if altered by an *ENG* mutation, could play a critical role in vascular pathologies, such as HHT [17]. Similarly, the conformational change in NAPG due to the identified mutation may cause a deficiency in platelet spreading by affecting its exocytosis, which in turn might influence vascular structures and result in HHT.

AVMs in HHT are characterized by a lack of intervening capillaries between arteries and veins. They may occur anywhere in the body and, due to their thin walls and proximity to the skin and mucosal surfaces, are prone to rupture and bleeding with minimal injury. Epistaxis and mucocutaneous telangiectasias are the most common clinical manifestations [3]. In addition, seizures caused by intracranial AVMs can occur in HHT [18, 19]. However, the incidence rate of intracranial AVM hemorrhage in patients with HHT is generally lower than that in patients with sporadic AVMs [19]. In our study, among the ten affected individuals with NAPG mutation, one exhibited no symptoms, seven exhibited both epistaxis and seizures, and two children in the fourth generation only exhibited epistaxis. HHT is an incomplete penetrance autosomal dominant multi-systemic disease, that exhibits increasing penetrance with age [20], which may explain why one affected individual exhibited no symptoms, and the two children only exhibited epistaxis.

Since HHT is a rare disease in the Chinese population, we have not encountered enough cases to verify the involvement of *NAPG* mutation in promoting HHT. Considering that the prevalence and genetic locus vary between different populations and regions, the association of this gene mutation and HHT needs to be validated in additional samples.

## Conclusions

This study used WES to identify a *NAPG* mutation in a Chinese family cluster of HHT. This gene mutation caused an amino acid change from methionine to valine, sabotaging the protein function by altering its structure, and probably promoting HHT. The discovery expands the current understanding of the genetic contribution to HHT pathogenesis.

## Supplementary Information

**Additional file 1**. The primers and methods used in the research.

## Data Availability

The datasets used and/or analyzed during the current study are available from the corresponding author on reasonable request.

## Abbreviations

HHT: Hereditary hemorrhagic telangiectasia
WES: Whole–exome sequencing
AVM: Arteriovenous malformations
MAF: Minor allele frequency
ESP: Exome Sequencing Project
ExAC: Exome Aggregation Consortium
HapMap: International Haplotype Map
ACVRL1: Activin A receptor-like type 1
ENG: Endoglin
SMAD: SMAD family member 4
NAPG: NSF attachment protein gamma
NHLBI: National Heart, Lung, and Blood Institute
