# Supplementary material for: NAPG mutation in family members with hereditary hemorrhagic telangiectasia in China
Source: BMC Pulm Med. 2021 Jun 10;21:197. doi: 10.1186/s12890-021-01524-4 (PMC8191015; doi:10.1186/s12890-021-01524-4)
Supplement: Supplementary file 1 — Additional file 1. The primers and methods used in the research. [file 12890_2021_1524_MOESM1_ESM.docx]

**Supplementary Table 1. The primers used for amplifying three known causal genes of HHT.**

| Name | Sequence |
| --- | --- |
| ACVRL1_1-f | 5' CAGGAAGACGCTGGAATAAGA 3' |
| ACVRL1_1-r | 5' CAGGTCACCCGACTAGAACC 3' |
| ACVRL1_2-f | 5' TCTCCACCCTTCACCTCT 3' |
| ACVRL1_2-r | 5' GCCTCACTGTCCTCCACT 3' |
| ACVRL1_3-f | 5' GAGGGACAGTAGGACAGAAATG 3' |
| ACVRL1_3-r | 5' CCAGACCACTCTGCCAGTTA 3' |
| ACVRL1_4-f | 5' CTCCCTTCCCTCCTTTCCT 3' |
| ACVRL1_4-r | 5' ACTGCAAGCTCCTCACTCG 3' |
| ACVRL1_5-f | 5' GTCCCAGGTCGAGGATAGAG 3' |
| ACVRL1_5-r | 5' CTGTGATTCCAGTAGCCAAAA 3' |
| ACVRL1_6-f | 5' CAGGGCTAGGTTCTTCTTTC 3' |
| ACVRL1_6-r | 5' GGTTCTGTTAATGTCTGGAGG 3' |
| ACVRL1_7-f | 5' CCACCCTGACCCTGACGACT 3' |
| ACVRL1_7-r | 5' GCGGAGGAGAACTGAAACAACA 3' |
| ACVRL1_8-f | 5' CGTCTCCATCTGCCTTCC 3' |
| ACVRL1_8-r | 5' TCTGACTGCAAACCTCCC 3' |
| ACVRL1_9-f | 5' CCTTGGATAGAGGGTAGAA 3' |
| ACVRL1_9-r | 5' GGGTTGAAAGAGGGAGTA 3' |
| ACVRL1_10-f | 5' CCATCCTCCTCATCTTCTTC 3' |
| ACVRL1_10-r | 5' CAGATAGGGCACCATCCA 3' |
| ENG_1-f | 5' TCCCAACGCCCACTGTTC 3' |
| ENG_1-r | 5' CCTCCACTCGACCCAGAAT 3' |
| ENG_2-f | 5' GCGAGTAGATGTACCAGAGTGC 3' |
| ENG_2-r | 5' TCCCGAGCCTCAGTTTCC 3' |
| ENG_3-f | 5' TGTCCTTTGTCCGCCTCT 3' |
| ENG_3-r | 5' GCTTCCTCCTCCACTTCTAC 3' |
| ENG_4-f | 5' TGTCCCTTCCTGCAAACC 3' |
| ENG_4-r | 5' AGGCACCGTGGAACTCAT 3' |
| ENG_5-f | 5' CGGCTAGAATTAAGAGTTCCC 3' |
| ENG_5-r | 5' AAGTCTCCCTCCCGTCCT 3' |
| ENG_6-f | 5' AGCGTCACCCTCAGCAGT 3' |
| ENG_6-r | 5' CATGCAGGTGTCAGCAAGTA 3' |
| ENG_7-f | 5' TGGTCAGAGCCAGAAAGG 3' |
| ENG_7-r | 5' CAGTGCCTCCTGATGGTG 3' |
| ENG_8-f | 5' TGTGGTCGGTGATGGACT 3' |
| ENG_8-r | 5' CTGCAACTGGCCTTCTTT 3' |
| ENG_9-f | 5' AAATACCAAGCCTCAATG 3' |
| ENG_9-r | 5' GGTTGTTCCCAATTCTGT 3' |
| ENG_10-f | 5' CTAAGGCTTGCAGAGGGA 3' |
| ENG_10-r | 5' CATGGGAACAATGGGAGT 3' |
| ENG_11-f | 5' TACCTTGCCCAAGCTCAC 3' |
| ENG_11-r | 5' CCCAAACTCCCAACCTCT 3' |
| ENG_12-f | 5' ATCCAGAGGTTGGGAGTTTG 3' |
| ENG_12-r | 5' GCCTGTCCGCTTCAGTGTT 3' |
| ENG_13-f | 5' TAGCCTAGAGTAGGAGAAAGCG 3' |
| ENG_13-r | 5' GGCAGGTGAGTGGTGGAA 3' |
| ENG_14-f | 5' AACCTGGCATATTCCACA 3' |
| ENG_14-r | 5' TTACTTCCTGACCTCCTACAT 3' |
| ENG_15-f | 5' TCAAGATGAAAGGGAGAAG 3' |
| ENG_15-r | 5' GTGGCACAACCTATACAAAT 3' |
| ENG_16-f | 5' GCCTTGGAGCTTCCTCTG 3' |
| ENG_16-r | 5' CTGGGTGTCCTTCTATTCATCG 3' |
| ENG_17-f | 5' CAGATTTCCAGAGGGCTAC 3' |
| ENG_17-r | 5' TACCAGGCACTCAACAGG 3' |
| ENG_18-f | 5' CCGAGGCTTTCTTTCAACAC 3' |
| ENG_18-r | 5' AGAAGGCTGGAGCAGGGAC 3' |
| SMAD4_1-f | 5' CCCTGAAATTACCCGGATGT 3' |
| SMAD4_1-r | 5' GGAAGGGGAGGGGACATG 3' |
| SMAD4_2-f | 5' ATCTTTTCCCAAGTAGTC 3' |
| SMAD4_2-r | 5' TTGCAAATGTTTCACTCT 3' |
| SMAD4_3-f | 5' CATGAATAAATGGTCGTT 3' |
| SMAD4_3-r | 5' CTTAGGATGAAAGCAAAG 3' |
| SMAD4_4-f | 5' GCGTTTATGCTACTTCTG 3' |
| SMAD4_4-r | 5' AACTGGCTCTGTGAGATT 3' |
| SMAD4_5-f | 5' CTGTTACCGCTGAATAAA 3' |
| SMAD4_5-r | 5' AGGAAAACTCAACTTGCT 3' |
| SMAD4_6-f | 5' AGTTTGCCTTTATAGATGAC 3' |
| SMAD4_6-r | 5' CAGGTGGTAGTGCTGTCT 3' |
| SMAD4_7-f | 5' CCAGGACAGCAGCAGAAT 3' |
| SMAD4_7-r | 5' AACAAAGCCCTACCAAAA 3' |
| SMAD4_8-f | 5' TGTACTGTATTGGACGTT 3' |
| SMAD4_8-r | 5' TCATCTGAGAAGTGACCC 3' |
| SMAD4_9-f | 5' TTTCTCATGGGAGGATGT 3' |
| SMAD4_9-r | 5' CACCGACAATTAAGATGGAG 3' |
| SMAD4_10-f | 5' ATTCATACTACATGCTCCTG 3' |
| SMAD4_10-r | 5' ATTCCTTCCACCCAGATT 3' |
| SMAD4_11-f | 5' AAGCCACCTTTCCTAACT 3' |
| SMAD4_11-r | 5' AAATGTCATCATCCCAGT 3' |
| SMAD4_12-f | 5' TAGGGAGGATGGGAAGAG 3' |
| SMAD4_12-r | 5' GGAGCAAGGCAGCAAACA 3' |

**Supplementary Table 2. The primers of 9 candidate genes identified from the HHT pedigree.**

| Primer | Sequence |
| --- | --- |
| ARAP3-f | 5' GGTAGACCTTGGCACCTTCCAAAGG 3' |
| ARAP3-r | 5' AAGCTGGCTGCCTCTTCA 3' |
| NAPG-f | 5' GCAGCATCCCTGGGTTCA 3' |
| NAPG-r | 5' TGGGTCCACTTACATCATTGTC 3' |
| C7orf50-f | 5' TAGTCCAGGGCCAGTTCG 3' |
| C7orf50-r | 5' GCTTAGCAGGATGGTGTCG 3' |
| CYP2W1-f | 5' CAGCACATCCACCCAGAGTC 3' |
| CYP2W1-r | 5' TCAGGGCGTCCACATAGC 3' |
| SRC-f | 5' GCTCCCTTCTCCTTTCCT 3' |
| SRC-r | 5' GCTCATTCCACAACACCC 3' |
| DKK2-f | 5' ATTGGCTCTGCCTCTTAT 3' |
| DKK2-r | 5' CTGAAAGCATCTTAACCC 3' |
| FNDC1-f | 5' ACAGTGTAATCAAGCCAGAC 3' |
| FNDC1-r | 5' CACTTACCCACATCCTCA 3' |
| GOLGA6L2-f | 5' CTCGGTAGAAGAATGGGATG 3' |
| GOLGA6L2-r | 5' GGGCAGGAGGAGAAGATG 3' |
| EIF3B-f | 5' GGATCACTTAGCAGCCTTGT 3' |
| EIF3B-r | 5' GTCATCCTGCGTGTCCAT 3' |

**Supplementary Table 3. Quality control of exome sequencing**

| Sample id | PCR duplication rate | | | | | Sequencing depth | | | | | | |
| --- | --- | --- | --- | --- | --- | --- | --- | --- | --- | --- | --- | --- |
|  | Un-paired Reads Examined | Read Pairs Examined | Mapped Reads | Percentage of Duplication | Estimate Library Size | Total reads | mean depth | %_bases_ above_1 | %_bases_ above_4 | %_bases_ above_10 | %_bases_ above_20 | %_bases_ above_50 |
| II-4 | 62,138 | 35,558,561 | 35,483,885 | 0.120603 | 632,398,920 | 5,065,859,600 | 67.99 | 99.4 | 98.9 | 97.1 | 90.2 | 56.5 |
| II-5 | 47,740 | 36,769,690 | 36,710,336 | 0.106778 | 874,101,113 | 5,600,429,004 | 75.16 | 99.5 | 98.9 | 96.6 | 89.4 | 59.9 |
| III-1 | 34,488 | 25,609,165 | 25,564,961 | 0.096258 | 682,067,530 | 3,838,436,165 | 51.51 | 99.4 | 98.7 | 94.9 | 82.9 | 41.5 |
| III-5 | 327,537 | 41,285,947 | 40,930,780 | 0.037814 | 815,068,435 | 4,518,513,538 | 60.64 | 99.4 | 98.9 | 97.3 | 90.7 | 51.1 |
| III-9 | 498,433 | 73,142,824 | 72,592,357 | 0.054097 | 865,063,478 | 8,116,664,347 | 108.93 | 99.5 | 99.3 | 98.7 | 96.9 | 82.1 |
| III-11 | 188,040 | 27,801,166 | 27,596,038 | 0.031866 | 717,702,279 | 3,196,768,271 | 42.9 | 99.4 | 98.6 | 94.5 | 80 | 30.8 |
| III-13 | 51,401 | 31,847,468 | 31,781,323 | 0.107152 | 688,593,190 | 4,711,426,714 | 63.23 | 99.3 | 98.7 | 96 | 87.5 | 52.4 |

**Supplementary Table 4. Free energy of amino acid residues.**

|  | Residue | Bonds | Angles | Torsion | Improper | Non-Bonded | Electrostatic constraint | Total |
| --- | --- | --- | --- | --- | --- | --- | --- | --- |
| NAPG p.262M>V | LEU258 | 3.722 | 6.151 | 10.276 | 0.052 | -33.84 | 6.45 | -7.197 |
|  | PHE259 | 6.886 | 26.992 | 4.514 | 4.807 | -46.08 | 10.66 | 7.785 |
|  | LYSH260 | 3.802 | 40523 | 3.942 | 20487 | -31.01 | -1.01 | -17.264 |
|  | TYR261 | 9.997 | 22.129 | 6.431 | 1.309 | -25.22 | -40.13 | -25.481 |
|  | VAL262 | 1.651 | 7.339 | 12.020 | 0.591 | -5.93 | 4.33 | **20.002** |
|  | ASP263 | 2.631 | 5.766 | 7.802 | 0.781 | -30.59 | 3.32 | -10.284 |
|  | ASN264 | 4.085 | 10.755 | 9.779 | 0.037 | -23.26 | -161.30 | -159.898 |
| NAPG wild-type | LEU258 | 4.229 | 6.706 | 10.232 | 0.038 | -34.93 | 6.30 | -7.426 |
|  | PHE259 | 5.900 | 24.369 | 5.222 | 4.985 | -48.72 | 8.89 | 0.646 |
|  | LYSH260 | 4.074 | 4.413 | 3.177 | 1.697 | -31.54 | -0.10 | -18.276 |
|  | TYR261 | 10.682 | 21.569 | 6.620 | 1.278 | -25.34 | -40.13 | -25.319 |
|  | MET262 | 5.655 | 9.500 | 14.429 | 0.151 | -43.43 | 4.00 | **-9.700** |
|  | ASP263 | 2.514 | 4.025 | 9.203 | 0.581 | -30.27 | 5.31 | -8.633 |
|  | ASN264 | 4.135 | 9.438 | 8.582 | 0.031 | -22.77 | -159.76 | -160.338 |

Bold fonts indicates free energy of the residues at position 262, before and after the *NAPG* c.784A>G mutation.
